# Genotoxic Damage During Brain Development Presages Prototypical Neurodegenerative Disease

**DOI:** 10.3389/fnins.2021.752153

**Published:** 2021-12-02

**Authors:** Glen E. Kisby, Peter S. Spencer

**Affiliations:** ^1^College of Osteopathic Medicine of the Pacific, Western University of Health Sciences, Lebanon, OR, United States; ^2^School of Medicine (Neurology), Oregon Institute of Occupational Health Sciences, Oregon Health & Science University, Portland, OR, United States

**Keywords:** neurodevelopment, genomic instability, cell-cycle re-entry, schizophrenia, amyotrophic lateral sclerosis, atypical parkinsonism, Alzheimer’s disease, Huntington’s disease

## Abstract

Western Pacific Amyotrophic Lateral Sclerosis and Parkinsonism-Dementia Complex (ALS/PDC) is a disappearing prototypical neurodegenerative disorder (tau-dominated polyproteinopathy) linked with prior exposure to phytogenotoxins in cycad seed used for medicine and/or food. The principal cycad genotoxin, methylazoxymethanol (MAM), forms reactive carbon-centered ions that alkylate nucleic acids in fetal rodent brain and, depending on the timing of systemic administration, induces persistent developmental abnormalities of the cortex, hippocampus, cerebellum, and retina. Whereas administration of MAM prenatally or postnatally can produce animal models of epilepsy, schizophrenia or ataxia, administration to adult animals produces little effect on brain structure or function. The neurotoxic effects of MAM administered to rats during cortical brain development (specifically, gestation day 17) are used to model the histological, neurophysiological and behavioral deficits of human schizophrenia, a condition that may precede or follow clinical onset of motor neuron disease in subjects with sporadic ALS and ALS/PDC. While studies of migrants to and from communities impacted by ALS/PDC indicate the degenerative brain disorder may be acquired in juvenile and adult life, a proportion of indigenous cases shows neurodevelopmental aberrations in the cerebellum and retina consistent with MAM exposure *in utero*. MAM induces specific patterns of DNA damage and repair that associate with increased tau expression in primary rat neuronal cultures and with brain transcriptional changes that parallel those associated with human ALS and Alzheimer’s disease. We examine MAM in relation to neurodevelopment, epigenetic modification, DNA damage/replicative stress, genomic instability, somatic mutation, cell-cycle reentry and cellular senescence. Since the majority of neurodegenerative disease lacks a solely inherited genetic basis, research is needed to explore the hypothesis that early-life exposure to genotoxic agents may trigger or promote molecular events that culminate in neurodegeneration.

## Developmental Origins of Neurodegenerative Disease

A developmental origin for neurodegenerative disorders, including amyotrophic lateral sclerosis with frontotemporal dementia (ALS/FTD), Huntington’s disease (HD), Parkinson’s disease (PD), Alzheimer’s disease (AD), and mild cognitive impairment (MCI), has been considered for many years (Gardener et al., 2010; Kovacs et al., 2014; Arendt et al., 2017; Ruzo et al., 2018; Wiatr et al., 2018; Kiernan et al., 2019; Starr, 2019; Lulé et al., 2020; Wang Z. et al., 2020), and recent clinical, genomic and molecular studies have uncovered evidence linking neurodevelopmental changes and neurodegenerative diseases. The onset of amyotrophic lateral sclerosis (ALS) may be related to neonatal dysfunction due to imbalances in excitation and inhibition (Kiernan et al., 2019), perhaps arising from the mutation of genes involved in neurodevelopment (i.e., *C9orf72*) (Yeh et al., 2018). Asymptomatic carriers of the *C9orf72* mutation in ALS/FTD exhibit white matter structural changes and cognitive decline that precede symptomatic disease onset, suggesting this is a neurodevelopmental disorder (Lulé et al., 2020). The fetal HD brain shows cortical malformations characterized by misaligned neurons, immature neurons, altered connectivity, multinucleated neurons and mitotic changes with altered cell-cycle progression likely due to chromosomal instability that occurred during fetal neurogenesis (Ruzo et al., 2018; Barnat et al., 2020). Neurodevelopmental changes in HD are responsible for age-related declines in cognitive function in children and adolescents (Schultz et al., 2021). Recent studies of neurons from subjects with familial and sporadic AD indicate that developmental processes are also consistently perturbed. Evidence from induced pluripotent stem cell-derived neurons from AD subjects indicates that diseased nerve cells develop features of de-differentiation reminiscent of a progenitor-like fate; they show reactivation of a cell-cycle phenotype, and they lack the resiliency of mature neurons (reviewed in Mertens et al., 2021). Collectively, recent studies of progressive neurodegenerative disorders suggest that genomic instability (i.e., mutations) during early brain development contributes to the pathogenesis of adult-onset neurodegeneration. Although familial progressive neurodegenerative diseases have provided clues about the role of toxic species of misprocessed peptides and proteins caused by specific mutations, sporadic forms of neurodegenerative disorders without clear evidence of a genetic etiology comprise the majority of cases. This suggests that environmental factors with potential developmental genotoxicity should be considered in the etiology and pathogenesis of sporadic forms of neurodegenerative diseases.

This review examines evidence that neuronal genome instability in the developing brain is impaired in sporadic forms of progressive neurodegenerative diseases. Particular focus is placed on the Western Pacific amyotrophic lateral sclerosis and Parkinsonism-dementia complex (ALS/PDC), a prototypical neurodegenerative disorder that finds clinical expression as ALS, atypical parkinsonism, dementia comparable to AD, and phenotypic mixtures thereof (Steele et al., 2010). Seven decades of research on ALS/PDC, principally on Guam where the disease was hyperendemic before slowly disappearing with post World War II modernization, have revealed in many cases fingerprints of disrupted brain development associated with exposure to specific genotoxic chemicals (Kisby and Spencer, 2011; Spencer et al., 2020a). A common theme among ALS/PDC and related neurodegenerative disorders is that cell-cycle changes and genomic instability occurring during brain development lead to the appearance and progression of brain disease in adulthood.

## Brain Development and Environmental Chemicals

Evidence is accumulating to indicate that early-life exposure to certain environmental chemicals is associated with neurological disorders (Grandjean and Landrigan, 2014). Exposure of the developing human brain to chemicals has been associated with a number of neurodevelopmental disorders, including epilepsy and schizophrenia. The timing of exposure (i.e., prenatal vs. postnatal) and the type of toxicant are two important factors that determine whether chemical exposure perturbs human brain development (Kisby et al., 2013; Heyer and Meredith, 2017; Bennett et al., 2019; Grandjean et al., 2019). There is experimental support for chemical-induced subthreshold perturbation of nigrostriatal neurons during vulnerable stages of neurogenesis, neuronal development and neuronal migration that bears on the susceptibility to PD in adult life (Barlow et al., 2007; Charlton, 2013; Schaefers and Teuchert-Noodt, 2016).

The prenatal period is characterized by the rapid expansion of proliferating neural stem/progenitor cells and the production and migration of immature neurons, whereas the postnatal period leads to the generation of glial progenitor cells and the maturation of neurons (Stiles and Jernigan, 2010). The prenatal period of human brain development, especially the first and second trimesters, is more vulnerable to environmental chemicals than the postnatal period (Heyer and Meredith, 2017; Fritsche et al., 2018) because neural progenitor cells are more sensitive to toxicants than glial cells or mature neurons (Druwe et al., 2015). Analysis of ALS/PDC has provided insight into how exposure to genotoxins (notably methylazoxymethanol, MAM) during critical periods of brain development may set the stage for latent neurodegenerative disease. Guided by observations from related neurodegenerative disorders, we examine prenatal, juvenile and young-adult exposure to MAM in regard to epigenetic modification, DNA damage/replicative stress, genomic instability, somatic mutation, cell-cycle reentry and cellular senescence.

## Environmental Etiology of Amyotrophic Lateral Sclerosis and Parkinsonism-Dementia Complex

Western Pacific ALS/PDC is a progressive neurodegenerative disease with multiple clinical phenotypes that has been highly prevalent in the island communities of the southern Marianas (Guam and Rota), Honshu, Japan (Kii Peninsula), and New Guinea (Papua, Indonesia). Given its often familial nature, inherited risk factors were first proposed, but with temporal decline in prevalence, some combination of genetic risk and environmental exposure was entertained. As disease rates continued to drop (Rodgers-Johnson et al., 1986), the dominant clinical presentation changed over time from ALS to parkinsonism-dementia (P-D) to Guam dementia (G-D), and the age of onset increased. It became increasingly clear that the decline of ALS/PDC on Guam was most probably associated with a disappearing environmental exposure associated with the acculturation of Chamorros to a Western lifestyle. Now that ALS has essentially disappeared from Guam, it is clear that the etiology of this formerly hyperendemic disease was primarily if not exclusively exogenous in origin (Giménez-Roldán et al., 2021).

Reduction in the incidence of ALS/PDC in all three geographic foci of the disease was associated with declining traditional use of a neurotoxic plant for food and/or medicine, specifically seed of the genus *Cycas*. The poisonous seed of *Cycas* spp. formerly served as a traditional food source for native Guamanians (Chamorros), an oral tonic and folk medicine in Kii-Japan, and a topical medicine for the treatment of open wounds in Papua-Indonesia, all of which have been linked to the subsequent development of ALS/PDC (Whiting, 1963, 1988; Kurland, 1972; Spencer et al., 1986, 1987a,b, 2005, 2020a,b,c; Spencer, 1987). Studies have shown that preference for traditional Chamorro food was significantly associated with an increased risk of P-D on Guam (Reed et al., 1975). Picking, processing and eating of cycad seed in young adulthood were consistently elevated and significant for dementia, MCI, and P-D on Guam (Borenstein et al., 2007). Additional information about the relationship between human exposure to cycads and ALS/PDC has been previously presented (Spencer et al., 2020a; Giménez-Roldán et al., 2021).

While *Cycas* seed contains a formidable mixture of chemicals, the two most studied are the neurotoxic amino acid β-*N*-methylamino-L-alanine (L-BMAA) and MAM, the aglycone of the plant glucoside cycasin (i.e., MAM-β-D-glucopyranoside). Analysis of cycad flour prepared Chamorro-style demonstrated the presence of cycasin and ten-fold-equivalent lower concentrations of L-BMAA (Kisby et al., 1992). Cycasin, but not L-BMAA, significantly correlated with the average annual age-adjusted incidence rates for ALS and P-D among Guamanian males and females (Román, 1996; Zhang et al., 1996). Cycasin also induces a motorsystem disease in ruminants that, in nature, results from eating young *Cycas* leaves (Shimizu et al., 1986; Spencer and Dastur, 1989). Cycasin is metabolized by β-glucosidases in plant and various tissues of animals and humans to MAM, a potent genotoxin and developmental neurotoxin that induces DNA damage both by oxidative stress (i.e., 8-oxoG) and the alkylation of guanine (i.e., *O*^6^-mG, N7-mG) (Kisby et al., 1999). Thus, MAM produces both oxidation- and alkylation-induced DNA damage, a property that might explain how human exposure to this genotoxin induces cell-cycle changes and genomic instability that lead to neurodevelopmental changes reported in ALS/PDC.

Evidence of exposure to a toxin early in development is suggested by the presence of ectopic, multinucleated, Purkinje-like cells in the cerebellum of Guam and Kii-Japan subjects who died of ALS/PDC in middle or late life (Shiraki and Yase, 1975; Yase and Shiraki, 1991; Morimoto et al., 2018). Guam ALS/PDC neurons with tau inclusions also contained mitotic markers (Husseman et al., 2000); such changes result from disruption of neuronal development, as seen in rodents treated with cycasin or MAM (Ferguson, 1996; Chevassus-au-Louis et al., 1999; Schwartzkroin and Wenzel, 2012; Kisby et al., 2013; Luhmann, 2016). While the age of ALS/PDC acquisition is unknown, exposure to cycads during childhood or young adulthood is a risk factor for G-D and PDC (Borenstein et al., 2007). These include retinal dysplasia (Spencer, 2020), hallmarks of which persist in adult life and predict the onset of ALS/PDC (Steele, 2008). Comparable lesions are produced in ferrets by single perinatal treatment with MAM (Haddad and Rabe, 1980).

## Cell-Cycle Changes in Development, Aging and Neurodegenerative Diseases

Cell-cycle dysregulation has been implicated in the pathogenesis of neurodevelopmental disorders (e.g., schizophrenia) (Benes, 2011), as well as neurodegenerative disorders like ALS, PD, and AD (Joseph et al., 2020; Zhang et al., 2021). Aberrant cell-cycle activation of post-mitotic neurons is a key molecular mechanism in AD, HD, ALS/PDC, and other human neurodegenerative disorders (Husseman et al., 2000; Lee et al., 2009; Fernandez-Fernandez et al., 2010; Moh et al., 2011). The cell cycle is a tightly orchestrated series of cellular events that leads to the duplication of genetic material. Preparation for cell division (G_1_ phase) is followed by DNA duplication (S phase), organization and condensation of genetic material (G_2_ stage), and cell division (M phase). Once mitosis is complete, neural cells exit the cell cycle and migrate to their respective positions in the developing brain. A balance of cellular proliferation and cell death mechanisms ensures cell and tissue homeostasis is maintained throughout human brain development. Disruption of this intricate network may result in a defective cell cycle that causes not only developmental changes but also neurodegeneration in later life (Park et al., 2007; Barrett et al., 2021).

Cell-cycle regulatory proteins in post-mitotic neurons are required for axonal migration, maturation and regulation of synaptic plasticity (Frade and Ovejero-Benito, 2015). While the post-mitotic neuron has exited the cell cycle (G_0_ phase) and undergone terminal differentiation, cell-cycle proteins can be reactivated in pathological states. However, instead of inducing cellular proliferation, neuronal reactivation results in aberrant cell-cycle reentry that culminates in protracted cell death (Xia et al., 2019). The expression of cell-cycle proteins is upregulated in post-mitotic neurons subjected to acute insults, such as growth factor deprivation, activity withdrawal, DNA damage, oxidative stress and excitotoxicity. After insult, neurons undergo abortive cell-cycle re-entry that is characterized by upregulation of cyclin-D-CDK4/6 activity and deregulation of E2F transcription factors that lead to neuronal apoptosis.

When subjected to stress, terminally differentiated neurons are susceptible to reactivation of their cell cycles and thereby become hyperploid. Several studies have noted that cell-cycle reentry in neurons leads to increased DNA levels (i.e., neuronal hyperploidy) (Frade and Ovejero-Benito, 2015) that is known to precede and recapitulate the classical neuropathological signs of AD (Yang et al., 2001; Arendt et al., 2010; Frade and López-Sánchez, 2017). Neuronal hyperploidy affects around 2–3% of neurons in AD (Mosch et al., 2007; López-Sánchez et al., 2017), a proportion that increases to around 8% when specific neuronal subtypes are evaluated (López-Sánchez et al., 2017). More than 30% of neurons become hyperploid in the middle stages of AD (Arendt et al., 2010) indicating that the fate of neuronal hyperploidy is delayed cell death (Yang et al., 2001; Arendt et al., 2010).

Neurons in the aging brain can also undergo cell-cycle re-entry that leads to DNA synthesis, with affected neurons remaining viable but with four complete chromosomes comprising double the normal DNA content (i.e., tetraploidy) (Frade and López-Sánchez, 2017). Injured nerve cells thus can actively re-enter the cell cycle, replicate their DNA, and survive as tetraploid neurons. Genetic instability associated with neuronal aneuploidy (more than diploid DNA content) also appears very early in the pathogenesis of AD, and these cells selectively die during the neurodegenerative process (Arendt et al., 2010). Forcing neurons to re-enter the cell cycle either by overexpressing an oncogene (Park et al., 2007; Barrio-Alonso et al., 2018; Barrett et al., 2021), or by treatment with a DNA-damaging agent (Zhang et al., 2020), is followed by hyperploidy, delayed death of neurons and/or induction of tau and amyloid pathology.

Remarkably, cell-cycle events can be maintained *in vivo* in affected neurons for weeks to years prior to apoptosis (which is regulated by E3 ubiquitin ligase Itch), suggesting that activation of the DNA-damage response (DDR) might be able to hold cell cycle-induced death (apoptosis) in check for prolonged periods (Lee et al., 2009; Ye and Blain, 2010; Chauhan et al., 2020). Cell-cycle dysregulation and misaligned neurons also occur in the fetal HD brain implying that these early events play a critical role in the ensuing pathogenesis of this neurodegenerative disorder (Barnat et al., 2020). Neuroprogenitor cells developed from iPSCs of ALS/FTD patients with the *C9orf72* mutation spontaneously re-express cyclin D after 3 months of differentiation into neurons, suggesting that cell-cycle re-entry (without apoptosis) plays an important role in the neuronal dysfunction in ALS and frontotemporal dementia (Porterfield et al., 2020). Thus, evidence of neuronal cell-cycle re-entry is commonly seen in age-related neurodegenerative diseases.

Many downstream factors of the DDR pathway promote cell-cycle re-entry in response to damage and appear to protect neurons from apoptotic death (Fielder et al., 2017). Post-mitotic neurons that survive the endogenous or exogenous DNA damage exhibit a persistent DNA-damage response, and they become senescent (Jurk et al., 2012; Fielder et al., 2017; Vazquez-Villaseñor et al., 2020). Instead of reacting to cellular/DNA damage by proliferation or apoptosis, senescent cells survive in a stable cell-cycle-arrest state (Sah et al., 2021). Senescent cells simultaneously contribute to chronic tissue degeneration by secreting deleterious molecules that negatively impact surrounding cells. It appears that cell-cycle re-entry, persistent DNA damage, and neuronal senescence are linked in age-related neurodegenerative diseases.

The ALS/PDC brain, like that of other tauopathies (AD, PSP, FTD linked to chromosome 17, Corticobasal Degeneration, Pick disease, Niemann Pick disease type C), shows markers of cell-cycle reactivation in neurons with tau pathology destined for degeneration (Husseman et al., 2000; Wang et al., 2009; Stone et al., 2011). The mitotic cdc2 kinase and its activator and cyclin B1 are found in degenerating neurons (tau phosphorylated at Thr231), as well as the cellular accumulation of phosphorylated MAP kinases (MAPK) that are known indices of cdc2 activity (Husseman et al., 2000). Hyperphosphorylated retinoblastoma protein (pRb), a cell-cycle G_1_-to-S phase checkpoint protein, is also elevated in Guam ALS/PDC neurons with and without neurofibrillary tangles (NFTs) (Stone et al., 2011). In non-dividing cells, the tumor suppressor protein pRb binds to E2F1, keeping it in an inactivated state (Zhang et al., 2020). One critical function of pRb is the control of the G_1_-to-S phase checkpoint of the cell cycle. In the hypophosphorylated state, the protein suppresses the activity of E2F transcription factors thereby inhibiting transcription of cell cycle-promoting genes. Upon phosphorylation, primarily by cyclin-dependent kinases, phosphorylated pRb dissociates from E2F and permits cell-cycle progression (Stone et al., 2011).

Evidence of cell-cycle perturbation early in development, in the form of hyperploid (i.e., bi- and tri-nuclear) and misaligned neurons, is evident in the cerebellum of both Guam and Kii-Japan ALS/PDC brains (Shiraki and Yase, 1975; Morimoto et al., 2018). Six of ten Japanese ALS/PDC patients had misaligned and multinucleated Purkinje cells in the molecular layer, and some of the misaligned Purkinje cells were positive for phosphorylated tau (serine 202/threonine 205). None of the controls had misaligned or multinucleated Purkinje cells. Moreover, in Japanese ALS/PDC brains, there is reduced expression of growth-arrest and DNA-damage/binding genes (e.g., *GADD-45* and *GADD-153*) (Morimoto et al., 2020). GADD-45 proteins have been associated with numerous cellular mechanisms including cell-cycle control, DNA-damage sensing and repair, genotoxic stress, neoplasia, and molecular epigenesis (Sultan and Sweatt, 2013). The misaligned and multinucleated neurons in the brains of Guam and Japanese subjects with ALS/PDC are consistent with aberrant cell-cycle re-entry during early brain development. Identification of predominant oxidative and nitrative DNA damage in Japanese ALS/PDC (Hata et al., 2018) is consistent with early-life exposure to a genotoxin. Collectively, these findings suggest that cell-cycle changes and DNA damage contribute to the underlying pathogenic mechanisms in ALS/PDC, possibly through a senescence type of mechanism.

## Cycad Genotoxins and Neurodevelopment

Exposure to cycad genotoxins during early human brain development might be an important driver of the ensuing pathological features of ALS/PDC, comparable to HD where a mutant gene (*Htt)* perturbs neurodevelopment that results in age-related decline in cognitive function (Ruzo et al., 2018; van der Plas et al., 2019; Barnat et al., 2020; Hickman et al., 2021; Schultz et al., 2021).

Long-term feeding (10 months) of a young (6-month-old) primate with chapati (baked pancakes) prepared from cycad flour provided by Guam residents resulted in weight loss, loss of hair, and anorexia by the 7th month, and liver damage (elevated liver enzymes) and severe weakness and wasting of muscles of one arm by the 9th month. The young primate also developed degeneration of motor neurons in both the motor cortex and spinal cord (Dastur, 1964; Dastur and Palekar, 1974). These neuropathological features were not observed in older primates that were similarly fed cycad-derived chapati. The liver of cycad-fed primates developed ‘large, irregular shaped bi- and trinucleated hepatocytes’ an indication that a genotoxin in cycad flour had disrupted liver cytokinesis (Shalakhmetova et al., 2009; Deng et al., 2011; Mukherjee et al., 2013) and, possibly, neuronal cytokinesis (*vide infra)*.

Cytokinesis is the final step in the cell cycle by which dividing cells physically separate into two cells following mitotic sister chromatid segregation. Multinucleation (the process of generating more than one nucleus) is a feature of neoplastic cells and reportedly due to abnormal cytokinesis or acytokinetic cell division through chronic activation of Akt, p53 loss, reduced expression of DNA repair genes or non-genetic aneuploidy (Mukherjee et al., 2013). Multinucleated cells are also commonly observed in the liver and brain of MAM-treated rodents (Jones et al., 1972; Zedeck et al., 1974; Borenfreund et al., 1975; Sullivan-Jones et al., 1994), and they can be experimentally induced in rodents by mutating genes “coding for proteins” that regulate cytokinesis (e.g., citron kinase, flathead, diaphanous) (Mitchell et al., 2001; Anastas et al., 2011; Harding et al., 2016) or by forcing cell-cycle re-entry in post-mitotic neurons (Walton et al., 2019); the latter results in hallmarks of AD, including neurofibrillary tangles, Aβ peptide deposits, gliosis, cognitive loss, and neuronal death (Barrio-Alonso et al., 2018, 2020). These phenotypic changes (i.e., multinucleation) are also characteristic features of the chromosomal instability observed in neurodegenerative diseases, which can result from defects in many aspects of the mitotic apparatus or unresolved DNA damage (Ruzo et al., 2018). Thus, it is reasonable to conjecture that cycad exposure of the young primate (*vided supra*) induced neuronal genomic instability and disrupted cytokinesis comparable to that observed in the brains of Guam and Kii Peninsula ALS/PDC subjects (Husseman et al., 2000; Stone et al., 2011; Morimoto et al., 2018).

The brains of some Japanese and many Guamanian subjects with ALS/PDC have multinucleated and ectopic Purkinje-like neurons in the cerebellum, with comparable developmental abnormalities of vestibular nuclei, occipital gyri and other areas of the brain (Yase, 1972; Shiraki and Yase, 1975; Yase and Shiraki, 1991; Morimoto et al., 2018). Comparable cerebellar dysplasia developed in early postnatal rodents following a single intraperitoneal injection of (the acetate form of) MAM (Shimada and Langman, 1970; Jones et al., 1972; Rabié et al., 1977; Lai et al., 1978; Haddad and Rabe, 1980; Bejar et al., 1985; Chen and Hillman, 1986, 1988, 1989; Yamanaka and Obata, 2004; Kisby et al., 2005, 2006, 2009, 2013). MAM disrupted cell division and migration that resulted in tissue disorganization featured by ectopic and misplaced Purkinje and granule cells (Shimada and Langman, 1970; Yamanaka and Obata, 2004). Neonatal administration of MAM perturbed cerebellar development in rodents such that, at 21 days of age, granule cells were mixed with Purkinje neurons instead of forming layers (Yamanaka and Obata, 2004; Schwartzkroin and Wenzel, 2012). Ectopic (heterotopic) neurons were also found in the hippocampus of neonatal rats following administration of MAM during fetal development (Singh, 1977). Based on human cerebellar development (Laure-Kamionowska and Maślińska, 2011), migrating granule and Purkinje cells would be at risk for MAM-induced disruption from the human second trimester onwards (Spencer, 2020).

Cycasin and MAM reproducibly induce pronounced changes in rodent brain that vary with the neurodevelopmental stage (Ferguson, 1996; Ferguson et al., 1996; Cattabeni and Di Luca, 1997; Colacitti et al., 1999). Rat pups treated with MAM on gestational day 15 (GD-15) or GD-17 develop neuropathological and behavioral changes consistent with focal cortical dysplasia (Paredes et al., 2006; Wong, 2009; Kim et al., 2017) or schizophrenia (Moore et al., 2006; Lodge and Grace, 2009; Du et al., 2020; Sonnenschein and Grace, 2020), respectively. A cortical dysplasia animal model can also be produced by administering MAM to pregnant ferrets (GD-33) resulting in reduced migration of cortical neurons (Schaefer et al., 2008) and increased GABA_A_ receptor expression (Abbah et al., 2014). Studies by Schaefer et al. (2008) suggest that the effect of MAM on the migration of cortical neurons in the GD-33 ferret model is due to reductions in *reelin*, a gene that undergoes epigenetic regulation and encodes a protein with an important role in the migration of cortical neurons in the brains of subjects with various psychiatric disorders (e.g., schizophrenia) (Ibi et al., 2020). Focal cortical dysplasia (FCD) leads to intractable epilepsy due to malformations of cortical development (Jesus-Ribeiro et al., 2021). Malformations of cortical development (MCD) result from the perturbation of different critical stages of human corticogenesis characterized by progenitor proliferation, neuronal migration, and connectivity (Subramanian et al., 2020). The neurodevelopmental changes in FCD are characterized by abnormal cortical structures and heterotopias caused by disruption of neuronal migration by somatic mutations of genes that encode proteins in the PI3K-AKT/mTOR pathway (Guerrini and Barba, 2021). Heterotopias are a common feature in both the GD-15 and GD-17 MAM animal models, and these pathological changes are comparable to the individual ectopic neurons reported in the brains of Guam and Kii ALS/PDC subjects (Shiraki and Yase, 1975; Morimoto et al., 2018) and HD (Hickman et al., 2021). Additionally, genome-wide association studies show that schizophrenia is associated with ALS (McLaughlin et al., 2017; Restuadi et al., 2021; Spencer and Kisby, 2021a), and diagnosis of schizophrenia prior to onset of motorsystem disease has been reported in ALS/PDC (Yase et al., 1972).

The MAM animal model of schizophrenia replicates changes both in mesolimbic dopamine function, which may contribute to the positive symptoms of schizophrenia, and to altered frontal cortical–limbic circuits thought to be associated with changes reminiscent of the negative and cognitive impairments of the human disorder (Jones et al., 2011). Schizophrenia-like deficits develop in the juvenile offspring of pregnant mice and rats treated with a carefully timed (GD-16 and GD-17, respectively) single dose of MAM (Lodge and Grace, 2009; Lodge, 2013; Dibble et al., 2016; Hu et al., 2018; Takahashi et al., 2019). This is accompanied by a reduced volume/weight of the hippocampus, entorhinal, parietal and prefrontal cortex and dorsal striatum, the first abnormalities associated with deficits in glutamatergic transmission and dopamine dysregulation in the prefrontal cortex and associated cognitive deficits (Featherstone et al., 2007; Matricon et al., 2010; Chin et al., 2011; Hradetzky et al., 2012; Lodge and Grace, 2012; Chalkiadaki et al., 2019; Takahashi et al., 2019). At 4 months of age, GD-17 MAM-treated rats develop schizophrenia-like features as indicated by enlarged lateral ventricles and altered cerebral blood flow (Drazanova et al., 2019), much like that observed in human schizophrenia (Kempton et al., 2010) and the frontal or temporal lobes of Kii ALS/PDC brains (Shindo et al., 2014). Larger lateral ventricular volumes have been associated with reductions in subcortical gray matter volume in schizophrenia (Horga et al., 2011). Adult rats exposed *in utero* to MAM also exhibit a significant reduction in neuronal spine density, as well as impaired working memory, changes that are blocked by treatment with a glycogen synthase kinase 3β (GSK3β) inhibitor during the juvenile period (Xing et al., 2018). GSK-3β (tau protein kinase 1) is implicated in the aggregation of hyperphosphorylated tau proteins into paired helical filaments that form NFTs in several neurodegenerative disorders, including ALS/PDC (Takashima, 2006; Kihira et al., 2009; Ma et al., 2017). In sum, there are links between prenatal exposure to MAM and the latent onset of abnormal brain structure and function. For humans, the second trimester is a period of risk for brain changes that result in childhood schizophrenia (Lodge and Grace, 2009).

## Epigenetic Changes and Cycad Toxins

Epigenetic modification can participate in many molecular biological processes, including gene expression, protein–protein interactions, cell differentiation, and embryonic development (Hwang et al., 2017; Berson et al., 2018). Histone modification, DNA methylation, and non-coding RNAs are three main epigenetic players that act on the PI3K-AKT-mTOR signaling pathway (Fattahi et al., 2020). The PI3K-AKT-mTOR pathway also plays an important role in cell-cycle re-entry and in blocking autophagy. If the epigenetic changes occur during critical periods of human brain development, they may lead to neurodegenerative disease. There is increasing evidence for the critical role of epigenetic changes in HD that are characterized by genome-wide DNA methylation and histone modification (Hyeon et al., 2021). Epigenetic modifications are also increasingly recognized to play a role in the etiology of schizophrenia (Khavari and Cairns, 2020; Bacon and Brinton, 2021; Richetto and Meyer, 2021) and progressive neurodegenerative diseases (Berson et al., 2018).

Prenatal administration of MAM to timed-pregnant rats (GD-17) disrupts epigenetic methylation of cytosine (i.e., DNA methylation) (Perez et al., 2016; Neary et al., 2017) during fetal development and, during postnatal life, alters the methylation of histone proteins (i.e., H3K4me3, H3K9me3, H3K27me3) and reduces the acetylation (i.e., H3K9ac) of histones in the prefrontal cortex of adults (Maćkowiak et al., 2014; Gulchina et al., 2017; Bator et al., 2018). In juvenile rats (PND-60) of the GD-17 MAM animal model of schizophrenia, Ingenuity pathway analysis revealed DNA methylation differences in hippocampal genes, changes in cell signaling, development and morphology that overlap with Neurological Diseases, Psychological Disorders and Developmental Disorders (Perez et al., 2016). Our KEGG pathway analysis of the differentially methylated genes in the hippocampus of these MAM-treated PND-60 rats revealed alterations in the PI3K-AKT/mTOR signaling pathway and pathways in cancer, similar to the DNA damage-anchored pathways seen in the whole brain of adult mice treated with a single systemic dose of MAM (Kisby et al., 2011). Since PI3K-AKT/mTOR regulates the cell cycle and cell proliferation/growth, its dysregulation is considered to have an important role in several neurodevelopmental diseases (e.g., microcephaly, schizophrenia, and epilepsy) (Wang et al., 2017) and in the neuronal cell-cycle changes observed in neurodegenerative disorders (Majd et al., 2019). Histone demethylation (H3Kme2) is also significantly reduced in the prefrontal cortex of neonatal (PND-15 and PND-45) rats treated with MAM on GD-17 (Maćkowiak et al., 2014), whereas histone trimethylation and histone acetylation are reduced the frontal cortex of adult animals (PND-60 and -70) that also had reduced expression of the gene encoding glutamic acid decarboxylase (*Gad1*) (Bator et al., 2018). Abnormal histone-mediated epigenetic silencing of the *Grin2b* gene is associated with *N*-methyl-D-aspartate receptor hypofunction in the premotor cortex of juvenile rats treated gestationally on day GD-17 with MAM, changes that may be related to cognitive impairment (Gulchina et al., 2017). Cell-cycle (*DP-1 and 2, CDK4, CDK6, RB, ORC6*) and chromatin-modifying genes (*HDAC4, HDAC9, HDAC11, JAR1D2, SMYD3*) are also differentially methylated in immature cortical neurons derived from human iPSCs following acute treatment (24 h) with 100 μM MAM (Spencer et al., 2020a). Furthermore, histone deacetylase (HDAC) I and II activity is significantly reduced in a concentration-dependent manner after acute treatment of human neuroprogenitor cells (hNPCs) with MAM. Thus, these studies demonstrate that, whereas histone acetylation is a late event, DNA methylation and histone methylation are early events following *in utero* treatment with MAM. Perhaps these epigenetic mechanisms explain how gestational exposure to MAM in the GD-17 rat induces postnatal, age-dependent GSK3β hyperactivity associated with significant reduction in dendritic spines, deficient long-term potentiation and facilitation of long-term depression in prefrontal cortical pyramidal neurons, together with working memory deficits (Xing et al., 2016).

## Genomic Instability and Cycad Toxins

The genomic stability of neurons is important for their development and maturation. Recent studies of HD provide clues about how early changes during *in utero* brain development impact the latent degeneration of neurons in this progressive genetic neurodegenerative disorder (Barnat et al., 2020). Examination of the brain of human fetuses (13 weeks of gestation) carrying mutant *Huntingtin* (*mHTT*) revealed abnormalities in the developing cortex caused by defects in neuroprogenitor cell polarity and differentiation, and changes in mitosis and cell-cycle progression. Barnat et al. (2020) also found that *mHTT*, in the 13-week-old human fetal brain and 13.5-day-old mouse embryonic brain, impaired the interkinetic nuclear migration of progenitors that caused premature commitment of neuronal precursors to their differentiated cell fate. These cortical malformations result from disruption at different critical stages of human corticogenesis—progenitor proliferation, neuronal migration, and connectivity (Subramanian et al., 2020). This developmental HD phenotype is characterized by giant multinucleated cortical neurons that are directly proportional to the length of the characteristic CAG trinucleotide repeat sequences, chromosomal instability and failed cytokinesis over multiple rounds of DNA replication during the neurogenesis of human cortical neurons (Ruzo et al., 2018).

**FIGURE 1:**
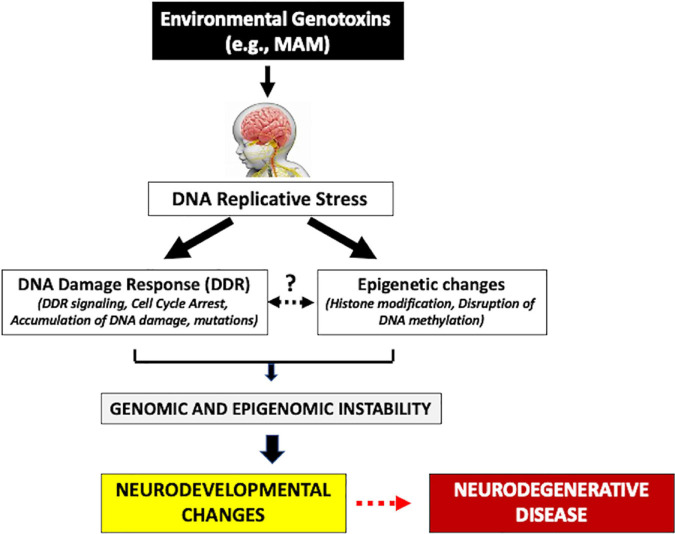
Human exposure to environmental genotoxins (e.g., methylazoxymethanol, MAM) during brain development (fetal/post-natal, childhood) may induce genomic/epigenomic instability that leads to sequential molecular changes culminating in later-life progressive neurodegenerative disease.

The mammalian target of the rapamycin (mTOR) pathway has been strongly linked with the underlying pathogenesis of these cortical malformations (Querfurth and Lee, 2021). The inhibition of the tuberous sclerosis protein complex (TSC), or activation of PIK3CA or AKT3, hyperactivates the mTOR pathway leading to dysregulated cell growth (Querfurth and Lee, 2021). Early alteration of the mTOR pathway also occurs in various proteinopathies (e.g., MCI, AD) (Tramutola et al., 2015). Such early neurodevelopmental and molecular changes may be important triggers of the neurodegeneration of HD and other latent progressive neurodegenerative diseases. The adult HD brain also shows various developmental malformations, including periventricular nodular heterotopia (PNH) (most frequent) and immature neuronal populations. PNH is characterized by altered neural migration and is usually associated with drug-resistant epilepsy and various psychiatric disorders (Fry et al., 2013; Jamuar et al., 2014; Alcantara et al., 2017; Ho et al., 2019). Interestingly, PNH can be experimentally reproduced following *in utero* injection of a genotoxin such as MAM or carmustine (Moroni et al., 2013; Luhmann, 2016) suggesting that early-life exposure to environmental genotoxins may be able to trigger similar molecular and neurodevelopmental changes. Heterotopias in the HD brain appear to be due to altered migration of cortical precursors through somatic mutations of migratory genes (Hickman et al., 2021). A mutation in *HTT* not only leads to a disruption of protein synthesis and progressive neurodegeneration, but these latent events are preceded by neurodevelopment changes during fetal brain development that disrupt the cell cycle, which can also be experimentally reproduced *in utero* by treatment of animals with genotoxic chemicals (Modgil et al., 2014; Koufaris and Sismani, 2015; Godschalk et al., 2020). As described above, cycad genotoxins are considered etiological agents of the prototypical progressive neurodegenerative disorder Western Pacific ALS/PDC (Spencer et al., 2020a). In sum, therefore, early life molecular and neurodevelopmental changes may be important triggers of latent progressive neurodegenerative disease (Figure 1).

### DNA Damage

While the role of cycasin/MAM, or its metabolites (such as formaldehyde) are individually, together, or with other factors, plausibly responsible for triggering ALS/PDC, it appears highly probable that these cycad toxins work primarily through the induction of genomic instability. MAM is widely known as a potent genotoxin that induces alkyl and oxidative DNA lesions (*O*^6^-mG, N7-mG, 8-oxoG) in many murine organs, including the brain (Inagake et al., 1995; Sohn et al., 2001; Kisby et al., 2011; Álvarez-González et al., 2015; Steullet et al., 2017). The oxidative DNA lesions (8-oxoG) observed in the brain of adolescent rats in the MAM GD-17 rat model most likely occurs via hydroxyradicals formed during autooxidation in the presence of metals such as iron (see Kuhnlein, 1980) or by inhibiting antioxidant enzymes (Azizi et al., 2018). While both DNA lesions occur in neurodevelopmental disorders and neurodegenerative diseases, interest focuses on *O*^6^-mG and 8-oxoG DNA lesions because they are pro-mutagenic for cycling cells and appear to be pro-cytotoxic in non-cycling cells, notably neurons (Larsen et al., 2006; Spencer et al., 2012).

Human and murine *O*^6^-alkyl lesions are subject to direct repair by *O*^6^-mG methyltransferase (MGMT) (Srivenugopal et al., 1996) or indirect repair by translesion synthesis repair (TLS) (Du et al., 2019). MGMT is effective at removing small alkyl groups from the *O*^6^-position of guanine (e.g., *O*^6^-mG), whereas repair of more bulky lesions relies on nucleotide excision repair. These lesions are highly mutagenic during cell replication and exclusively direct G to A mutations with participation by DNA polymerase pol eta and pol gamma (error-prone bypass) of the small alkyl lesions, whereas *pol *kappa** incorporates the correct base (i.e., dCMP) opposite the lesion. DNA polymerases and replication factor c, which are involved in TLS (i.e., pol kappa, pol delta), are among the genes differentially methylated in the hippocampus of MAM-treated adolescent rats (see Supplementary Table S1 in Perez et al., 2016). Failure to repair *O*^6^-mG lesions in cells undergoing division increases the risk of mispairing with thymine during DNA replication, resulting in GC:AT transitions and frameshift mutations in bacteria (Hoffmann et al., 2002). MGMT enzyme activity is especially required during cell division but, in post-mitotic nerve cells, activity appears to be very low, such that neurons should be highly susceptible to MAM. Given the low capacity of brain vs. liver tissue to repair *O*^6^-mG lesions (Kleihues and Bucheler, 1977; Wiestler et al., 1984; Silber et al., 1996), repeated exposure to alkylating agents such as MAM would result in mounting DNA damage and genomic instability (Kisby et al., 1999, 2011).

Oxidative DNA damage (i.e., 8-oxoG) has also been observed in the brain of rodents following early-life exposure to MAM (Steullet et al., 2017); this type of DNA damage is also repaired by TLS, which results in the correct insertion of a C opposite the 8-oxoG (Markkanen et al., 2012). These studies demonstrate that MAM indirectly induces oxidative DNA damage in neural tissues through a transition metal-catalyzed mechanism (see Kuhnlein, 1980) or by reducing antioxidant enzymes (Azizi et al., 2018).

Recent studies show that the DNA damage induced by genotoxins in post-mitotic neurons maps to hotspots of DNA repair across the genome (Reid et al., 2021; Wu et al., 2021), with repair sites predominantly located in neuronal enhancers at sites of CpG DNA methylation (Wu et al., 2021). Such mechanisms may explain the elevated levels of DNA damage and epigenetic changes that occur in the prefrontal cortex of the MAM animal model of schizophrenia (Maćkowiak et al., 2014; Perez et al., 2016; Gulchina et al., 2017; Steullet et al., 2017) and the neurodegenerative phenotype that develops in inherited DNA-repair disorders (Tiwari and Wilson, 2019; Ainslie et al., 2021; Gupta et al., 2021).

### DNA Replicative Stress

In response to endogenous or exogenous DNA damage, cells rapidly activate DNA damage response (DDR) mechanisms to remove and repair lesions by specific DNA-repair pathways and coordinate these events with cell-cycle progression and apoptosis (Ciccia and Elledge, 2010; Polo and Jackson, 2011; Iyama and Wilson, 2013). DDR is achieved through the simultaneous collaborative actions of multiple checkpoint and repair proteins that detect the damage and remodel the chromatin coordinating, the repair with cell-cycle progression, inducing apoptosis, autophagy, or senescence if the damage is left unrepaired (Harper and Elledge, 2007; Soria et al., 2012). During neurodevelopment, maintaining the integrity of DNA is critical for preventing DNA damage, mutations and faithfully transferring epigenetic information (Tsegay et al., 2019). If DNA damage occurs during brain development, it can alter the accuracy of DNA replication (e.g., cell-cycle changes), integrity and epigenetic features, resulting in DNA-replication stress and genome and epigenome instability (Fielder et al., 2017; Wezyk et al., 2018).

Several exogenous and endogenous agents induce replication stress, including DNA lesions or adducts caused by chemical compounds, ultraviolet or ionizing radiation, reactive oxygen species, byproducts of cellular metabolism, nucleotide pool imbalance or a shortage of replication factors (Mazouzi et al., 2014; Zeman and Cimprich, 2014). DNA damage-induced replication stress reportedly plays an important role in neurodevelopmental disorders (Wang M. et al., 2020) and neurodegenerative disease (Wilhelm et al., 2020). DNA damage is observed in several genes vulnerable to replication stress in neural progenitor cells derived from patients with autistic spectrum disorder (ASD) (Wang M. et al., 2020). Neuroprogenitor cells (NPCs) derived from induced pluripotent stem cells of these patients exhibit accelerated S-phase progression (cell-cycle changes), increased DNA replication stress, and chronic DNA damage when compared to NPCs derived from control subjects. DNA replication stress attenuates gene expression involved in adherens junctions, apical polarity, cell migration and other NPC functions. Exome sequencing of ASD hNPCs reveals mutations in several genes in the canonical Wnt pathway, cell-cycle regulation, mitotic checkpoints, and DNA repair, as well as in genes that maintain genomic stability (*ATM, BRACA1 CDK7, ERCC4*). These recent studies indicate that DNA damage-induced replication stress is a key underlying mechanism of autism and possibly other neurodevelopmental disorders (Charlier and Martins, 2020). DNA damage-induced replication stress might also be an important mechanism to explain how early-life exposure to environmental genotoxins induces long-term effects that lead to neurodegenerative disease.

### Cellular Senescence

How does human exposure to the cycad genotoxins cycasin/MAM induce a progressive neurodegenerative disorder like ALS/PDC? The answer may lie in the ability of these genotoxins to induce cellular senescence in neurons; this is reportedly caused by epigenetic changes, teleomere attrition, DNA damage and mitochondrial dysfunction that culminate in dysfunction of nutrient signaling and proteostasis (Sherman et al., 2011; Fielder et al., 2017; Vazquez-Villaseñor et al., 2020; Gillispie et al., 2021). The most widely accepted phenotype of a senescent cell is a change in cell fate that accompanies cell-cycle arrest, like that seen in human neuroprogenitor cells treated with MAM (Spencer et al., 2020a). Another characteristic feature of a senescent neuron is that it develops morphological and functional changes, notably increased cell size and altered nuclei that are either enlarged (karyomegaly) or multinucleated. These features are also characteristic of neurons or non-neuronal rodent cells (e.g., liver) after treatment with MAM (Zedeck et al., 1974; Borenfreund et al., 1975; Sullivan-Jones et al., 1994).

A senescent phenotype in rodent and human neural stem cells (NSCs) has been observed in response to chemical carcinogens (Dong et al., 2014; Daniele et al., 2016). NSCs exist primarily in a quiescent state by lowering metabolic activity and cell division to minimize damage to DNA, proteins, and mitochondria, which can lead to tumorigenesis, senescence and depletion of the stem cell pool. Senescence may protect NSCs from becoming cancerous in response to a carcinogen (Campisi, 2013) through activation of this stress response to promote short-term health and survival. Mouse and human NSCs treated with the genotoxin hydroxyurea show a senescent like phenotype that includes reduced proliferation, p21, p16, and increased DNA damage via p53. A reduction in apoptosis in hydroxyurea-treated NSCs derived from rats is also characterized by the activation of NF-kB and other cell signaling pathways (i.e., p38/MAPK, ERK1/2) (Dong et al., 2014) that are also targeted by MAM and anchored to DNA lesions in mice (Kisby et al., 2011). Cellular senescence is emerging as a potentially important pathway for understanding the long-term effects of genotoxin-induced DNA damage on both neurodevelopment and neurodegeneration.

Senescent neurons are also characterized by the co-expression of cell-cycle mediators in the absence of apoptotic markers (Sah et al., 2021). In response to injury (or DNA damage-induced stress), mitotically competent cells may proliferate (e.g., tumorigenesis) whereas post-mitotic cell-cycle re-entry triggers a degenerative process. Cell-cycle re-entry has been estimated to occur in ∼11.5% of post-mitotic cortical neurons via DNA content variation and 20% expression of post-mitotic neurons in AD through both DNA content variation and expression of cyclin B. These data indicate an important link between neuronal cell-cycle activity, neuronal dysfunction and neurodegenerative disease. The cycad genotoxins cycasin/MAM induce genomic instability most likely through DNA damage and epigenetic mechanisms that cause stable cell-cycle arrest in neurons.

### Somatic Mutations

Another important question is whether the cycad genotoxins cycasin/MAM can induce persistent neuronal DNA damage that could lead to an increased mutational burden of nerve cells. We have shown that alkyl DNA damage (i.e., *O*^6^-mG) remains elevated in brain tissue of MAM-treated young adult mice when levels in the liver have declined, a result indicating that the DNA damage persists in the rodent brain because of inefficient repair (Kisby et al., 2011). Oxidative DNA lesions produced by MAM have also been shown to persist in the brain of DNA repair (xeroderma pigmentosum A, XPA)-deficient mice (Mori et al., 2019). XP-A patients have the most severe and earliest forms of the neurological disorder with premature aging features of peripheral neuropathy, progressive sensorineural hearing and neurodegeneration (Rizza et al., 2021). The lack of predominant neuronal cell death in the face of elevated alkyl DNA damage in the brain of MAM-treated mice is consistent with their persistence and inefficient removal by rodent brain DNA-repair processes (Margison and Kleihues, 1975; Buecheler and Kleihues, 1977; Kleihues and Bucheler, 1977; Kleihues et al., 1980). Moreover, the 60 genes that were anchored to the MAM-induced DNA lesions (*p53*, *NFk-B*, *MAPK*) reported by Kisby et al. (2011) play an important role in DNA damage-induced senescence in neurons (Dong et al., 2014). Therefore, the alkyl and oxidative DNA lesions produced by the cycad genotoxin MAM have been shown to accumulate in the brain of rodents, which suggests their persistence in neurons could lead to somatic mutations.

The increased dependency on DNA-repair synthesis at specific sites of the genome also increases the mutational burden in long-lived neurons (Lodato et al., 2018). Three mutational signatures are found in long-lived human neurons: a post-mitotic, clock-like signature of aging, a possible developmental signature that varies across brain regions, and a disease- and age-specific signature of oxidation and defective DNA-damage repair (termed genosenium). It is possible that mutations could accrue at sites of recurrent DNA-repair synthesis within the neuronal genome that could lead to aberrant gene expression, resulting in neurological dysfunction and progressive neurodegeneration. A common thread between neurodevelopmental disorders and neurodegenerative disease is that long genes are prone to somatic mutations (Larsen et al., 2016; Gozes, 2021; Soheili-Nezhad et al., 2021). Long genes are also more prone to DNA damage because DNA repair appears to be less efficient for neuronal genes above 500K bp (Reid et al., 2021). Therefore, efficiency of DNA repair is essential for maintaining genomic stability and preventing genotoxin-induced mutations in neurons during brain development (McKinnon, 2017). The cycad genotoxin MAM might induce somatic mutations in the developing brain through a similar mechanism (Spencer and Kisby, 2021a).

The association between mutational spectra and their underlying mutagenic processes is complicated as mutations arise from various DNA lesions, which are repaired by numerous and partially redundant DNA-repair pathways (Volkova et al., 2020). As neurons age, the activity of DNA-repair mechanisms declines, leading to an increase in somatic mutations and the accumulation of unrepaired lesions (Maynard et al., 2015). Hence, there are at least two unknowns that contribute to a mutational spectrum: DNA damage and DNA repair. The fact that these counteracting processes jointly shape genotoxin-induced mutagenesis is perhaps best exemplified by the interplay of nucleotide misincorporation by replicative DNA polymerases and mismatch repair (MMR) (Volkova et al., 2020). MMR operates downstream of the replication fork and repairs many mismatches caused by misincorporated nucleotides, often in a base-specific way. If polymerase fidelity is compromised, MMR provides a backstop and observed mutations stem from those lesions (e.g., *O*^6^-mG, 8-oxoG) that escaped MMR or were incorrectly repaired; the full spectrum of replication errors only becomes visible under MMR deficiency. Both of these processes are involved in repairing the DNA lesions produced by MAM (Volkova et al., 2020).

Somatic mutations arising from the brain have recently emerged as significant contributors to neurodevelopmental disorders, including focal cortical dysplasia, cortical malformations and schizophrenia (Baldassari et al., 2019; Kim et al., 2021). Using advanced genetic tools and sequencing coverage, surgical brain tissue that was isolated from patients with FCD showed that 60% of them had brain somatic mutations in the mTOR pathway, a pathway that plays an important role in cortical migration (Baldassari et al., 2019; Guerrini and Barba, 2021) and is targeted by MAM in the GD-17 animal model of schizophrenia (Perez et al., 2016).

The mutational signatures for 79 genotoxic chemicals were recently evaluated in human iPSC cultures after controlling for mutagenesis by reactive oxygen species (Kucab et al., 2019); alkylating agents showed similar and unique mutational signatures. 1,2-Dimethylhydrazine (DMH) and temozolamide are two genotoxins with a MAM-like DNA lesion profile that were evaluated for mutational signatures. DMH was incubated with S9 liver extract to convert the substance into MAM, its mutagenic metabolite (Fiala, 1975, 1977). DMH produced *de novo* single substitution mutations and deletions in human iPSC cells (Kucab et al., 2019). The DMH *de novo* substitutions were due to alkylation of guanines to *O*^6^-mG (i.e., ApG sites) whereas the deletions (loss of T) were due to ‘slippage’ following replication of a G > A substitution. The C > G deletions by DMH are likely due to slippage by mismatch DNA repair, which commonly occurs in colorectoral carcinomas (Kunkel and Erie, 2005). Despite these mutational changes, there was high chromosomal stability due to efficient DDR and cell-cycle checkpoint activity in the human iPSCs subclones. Since these studies utilized normal human iPSCs, they might not reflect the mutational signatures in neural cells derived from genotoxin-derived iPSCs. Such studies could reveal the importance of early genotoxin-produced mutations (i.e., timing of exposure) on neurodevelopment as well as the neurodegenerative disease process.

## Summary and Conclusion

Among the plethora of neurodegenerative disorders, Western Pacific ALS/PDC is uniquely important for several reasons (Spencer et al., 2020a): (a) its etiology is primarily or exclusively exogenous; multiple studies have failed to reveal any consistent genetic component; (b) a large body of clinical and experimental data point to an etiologic role for genotoxins, notably cycasin and its active metabolite MAM; (c) traditional use on Guam of cycad-derived food products containing cycasin/MAM indicates that exposure would have occurred at all stages in life, including *in utero*; (d) animal studies show that perinatal MAM treatment induces cerebellar and retinal dysplasia, clinical studies show that the anatomical hallmarks of retinal dysplasia predict risk for ALS/PDC, and neuropathological examination of some cases reveals evidence of ectopic and multinucleated Purkinje cells, which corresponds to neurodevelopmental changes that occurred in the second half of pregnancy. Unknown is whether, in these cases, the developmental changes time the trigger for onset of the pathological process that culminates in ALS/PDC. Epidemiological and migration studies reveal cases that acquired risk for ALS/PDC as children or young adults, although clinical evidence of disease may not appear until many decades later. ALS usually appeared in younger adults, P-D at later ages, and AD-like dementia (G-D) in the oldest Guamanians, a phenotypic sequence suggestive of a dose-response pattern, the highest exposure to cycad toxins resulting in ALS and the lowest in Guam Dementia.

While MAM may induce neuronal changes prior to and following terminal cell division, of critical importance is that neurons do not undergo apoptotic cell death but rather show evidence of progressive disease. Guam ALS/PDC neurons with tau inclusions contain mitotic markers (Husseman et al., 2000; Stone et al., 2011) indicative of abnormal neuronal development, as occurs in neonatal rodents treated with cycasin or MAM (Sullivan-Jones et al., 1994; Ferguson, 1996; Kisby et al., 2013). These findings are consistent with emerging evidence indicating that neurons in the brains of HD, AD and other neurodegenerative diseases exhibit features of genomic instability, notably binucleation, cell-cycle disturbances, and aneuploidy (Lee et al., 2009; Fernandez-Fernandez et al., 2010; Moh et al., 2011).

Work on the etiology of Western Pacific ALS/PDC and related tauopathies raises some important questions. Is the developing brain more vulnerable to cycad genotoxins (cycasin, MAM) than the mature brain? Do the changes induced by cycad genotoxins (e.g., DNA damage-induced replicative stress, senescence, somatic mutations) in the developing brain herald the onset of changes that culminate in neurodegenerative disease? Similar ideas have been discussed in relation to other progressive neurodegenerative disorders, such as AD (Vincent et al., 2003; Bajić et al., 2009). Answers to these questions are likely to provide further insight into the underlying mechanisms of ALS/PDC and related progressive neurodegenerative diseases.

Finally, as discussed elsewhere, the molecular mechanisms utilized by MAM are shared by other genotoxic chemicals, including nitrosoureas, nitrosoamines, and hydrazines (Spencer and Kisby, 2021b), exposure to which has also been associated with neurodegenerative disease, notably sporadic ALS (Spencer, 2019; Lagrange et al., 2021). This should encourage intense study of the lifetime exposure history of subjects with non-inherited neurodegenerative disorders that, like ALS/PDC, may be triggered by environmental genotoxins.

## Author Contributions

Both authors contributed to the article and approved the submitted version.

## Conflict of Interest

The authors declare that the research was conducted in the absence of any commercial or financial relationships that could be construed as a potential conflict of interest.

## Publisher’s Note

All claims expressed in this article are solely those of the authors and do not necessarily represent those of their affiliated organizations, or those of the publisher, the editors and the reviewers. Any product that may be evaluated in this article, or claim that may be made by its manufacturer, is not guaranteed or endorsed by the publisher.

## Abbreviations

AD: Alzheimer’s disease
ALS: amyotrophic lateral sclerosis
ALS/FTD: amyotrophic lateral sclerosis/frontotemporal dementia
ALS/PDC: amyotrophic lateral sclerosis and Parkinsonism-dementia complex
ASD: autism spectrum disorder
DDR: DNA damage response
DMH: 1,2-dimethylhydrazine
FCD: focal cortical dysplasia
GADD: growth-arrest and DNA-damage/binding protein
GD: gestational day
G-D: Guam dementia
GSK3 β: glycogen synthase kinase 3 β
HD: Huntington’s disease
HDAC: histone deacetylase
*HTT*: *Huntingtin* gene
hNPC: human neuroprogenitor cell
iPSC: induced pluripotent stem cells
KEGG: Kyoto encyclopedia of genes and genomes
MAPK: mitogen-activated protein kinase
MCD: malformations of cortical development
MCI: mild cognitive impairment
MGMT: *O*^6^-mG methylguanine methyltransferase
MMR: mismatch DNA repair
NFT: neurofibrillary tangle
NPC: neuroprogenitor cell
NSC: neural stem cell
*O*^6^-mG: *O*^6^-methylguanine DNA adduct
PD: Parkinson’s disease
PND: postnatal day
PNH: periventricular nodular heterotopia
PSP: progressive supranuclear palsy
pRb: tumor suppressor protein retinoblastoma
TLS: translesion synthesis repair
TSC: tuberous sclerosis protein complex.
